# Implicit manifestation of prospective metacognition in betting choices enhances its efficiency compared to explicit expression

**DOI:** 10.3389/fnhum.2025.1490530

**Published:** 2025-03-05

**Authors:** Hidekazu Nagamura, Hiroshi Onishi, Kohta I. Kobayasi, Shoko Yuki

**Affiliations:** ^1^Graduate School of Life and Medical Sciences, Doshisha University, Kyoto, Japan; ^2^Graduate School of Arts and Sciences, The University of Tokyo, Tokyo, Japan

**Keywords:** prospective metacognition, metacognitive efficiency, metamemory, wagering, confidence rating

## Abstract

Recent metacognitive research has extensively investigated metacognitive efficiency (i.e., the accuracy of metacognition). Given the functional importance of metacognition for adaptive behavioral control, it is important to explore the nature of prospective metacognitive efficiency; however, most research has focused on retrospective metacognition. To understand the nature of prospective metacognition, it is essential to identify the factors that influence its efficiency. Despite its significance, research exploring the factors of prospective metacognitive efficiency remains scarce. We focused on the relationship between the efficiency of prospective metacognition and the manner in which metacognition is inferred. Specifically, we explored whether explicit metacognition based on verbal confidence reports and implicit metacognition based on bets produce differences in efficiency. Participants were instructed to either respond to a memory belief with a sound (explicit metacognition) or make a bet on its recallability (implicit metacognition) during a delayed match-to-sample task. The task was identical for all participants, except for the pre-rating instructions. We found that the efficiency of prospective metacognition was enhanced by the betting instructions. Additionally, we showed the possibility that this difference in metacognitive efficiency was caused by the difference in pre-rating variability between the instructions. Our results suggest that the way a person evaluates their own internal states makes the difference in the efficiency of prospective metacognition. This study is the first to identify a factor that regulates the efficiency of prospective metacognition, thereby advancing our understanding of the mechanisms underlying metacognition. These findings highlight that the potential influence of framing, such as instruction, can improve metacognitive efficiency.

## 1 Introduction

### 1.1 Metacognition

In an uncertain environment, individuals utilize not only information from the external world, but also internal states such as confidence, ease of memory retention, and prediction of the likelihood of recall to achieve flexible behavioral control (Hart, 1965; Arbuckle and Cuddy, 1969; Nelson and Louis, 1990). For example, even if there is no external feedback on an action, people can appropriately adjust their behavior by referring to their internal states. This function is called metacognition and often described by two major processes, monitoring and control (Flavell, 1979; Nelson and Louis, 1990).

Commonly known as the “two-process model,” this framework suggests that monitoring entails evaluating and understanding one’s own cognitive states, while control involves modifying behavior or thought processes based on the outcomes of monitoring (Flavell, 1979). For instance, if monitoring indicates that a piece of information is not well retained, an individual might engage in additional study or adopt a more effective learning strategy. This continuous interplay between monitoring and control forms the foundation of adaptive metacognitive functioning, enabling individuals to refine their actions, even without explicit external feedback. By continually assessing and regulating cognitive processes in this manner, people can maximize their performance in uncertain or changing environments and maintain flexible behavioral control (Gourgey, 1998; Egner and Siqi-Liu, 2024).

### 1.2 Metacognitive efficiency

Recent studies have particularly focused on “metacognitive efficiency” (Maniscalco and Lau, 2012; Fleming and Lau, 2014; Fleming, 2017). Metacognitive efficiency refers to the accuracy of metacognition corrected with perceptual/memory performance, and is considered crucial for real-life applications. For instance, Fischer et al. (2023) reported that individuals with higher metacognitive efficiency are better able to make appropriate judgments about risks, such as vaccinations during the COVID-19 pandemic. Accurate metacognition facilitates flexible, risk-based decision-making; conversely, inaccurate metacognition can result in suboptimal decisions and may aggravate psychiatric symptoms. Notably, impaired metacognitive processes have been associated with various psychiatric conditions, such as schizophrenia and depression, where individuals struggle with accurate self-monitoring and judgment (Wells, 2011; Hasson-Ohayon et al., 2015; Martiadis et al., 2023; Favaretto et al., 2024). These findings indicate that enhancing metacognitive efficiency may promote better mental health outcomes and support adaptive functioning in real-life contexts.

### 1.3 The efficiency of prospective and retrospective metacognition

Metacognition can be conceptualized along a temporal dimension, with prospective metacognition focusing on future performance and retrospective metacognition examining past performance (Fleming et al., 2016; Siedlecka et al., 2016). Prospective metacognition is particularly significant for planning, decision-making, and anticipating risks, while retrospective metacognition aids individuals in reflecting on and learning from past outcomes (Fleming and Dolan, 2012; Siedlecka et al., 2016). Despite increasing interest in prospective metacognition (Fleming, 2024; Katyal and Fleming, 2024), most research on metacognitive efficiency has predominantly centered on retrospective processes (Fleming and Dolan, 2010; Goupil and Kouider, 2016; Pereira et al., 2020).

Some comparative studies of prospective and retrospective metacognition have shown that retrospective metacognition has a stronger positive correlation with task performance only after the motor response than prospective metacognition, but not before (Siedlecka et al., 2016, 2019; van den Berg et al., 2016; Qiu et al., 2018). These observations suggest that retrospective metacognition attains greater accuracy by integrating external cues, such as motor information, rather than relying exclusively on internal states. As a result, investigating the efficiency of prospective metacognition offers a meaningful pathway for enhancing our understanding and evaluation of internal-state monitoring accuracy.

### 1.4 The factor influencing metacognitive efficiency

Previous research has demonstrated that retrospective metacognition is shaped by factors such as response feedback, priming with task information, and task difficulty (Zohar and Barzilai, 2013; Desender et al., 2017; Carpenter et al., 2019; Luo and Liu, 2023). For instance, Carpenter et al. (2019) investigated whether metacognitive efficiency could be systematically enhanced through adaptive training in a perceptual discrimination task. Their findings revealed that participants who received feedback on their confidence-accuracy relationship demonstrated improvements in metacognitive efficiency without corresponding changes in perceptual or memory performance. Moreover, these benefits were transferred to both trained and untrained recognition memory tasks. However, these effects are minimal and short-lived (Rouy et al., 2022). In a subsequent analysis, Rouy et al. (2022) revisited Carpenter et al.’s study, suggesting that the original findings might have been confounded by inconsistencies in incentives and confidence scales. After replicating the study under more controlled conditions, they found no evidence of genuine metacognitive improvement and concluded that more rigorous experimental designs are required to determine whether metacognitive efficiency can be systematically trained.

Prospective metacognition has been studied using various measures including judgments of learning (JOL), predictions of future recall performance, and feelings of knowing (FOK), subjective experiences of knowing information that cannot currently be recalled (Hart, 1965; Nelson and Louis, 1990; Koriat, 1997). Despite progress in characterizing prospective metacognition through these measures, our understanding of the factors governing its efficiency remains limited. For example, Koriat (1997) demonstrated that the accuracy of JOLs is sensitive to the inherent difficulty of memory items but not to external factors such as exposure duration or frequency. While the accuracy of metacognition is known to be influenced by actual behavioral performance (Maniscalco and Lau, 2012; Fleming and Lau, 2014), prospective metacognitive research using JOLs and FOKs has often overlooked the impact of memory performance itself on the efficiency of these judgments. This oversight may restrict our understanding of prospective metacognitive efficiency.

### 1.5 Functional differences between explicit and implicit metacognition

In this study, we focus on task instructions in which the internal state was intended to be systematically controlled. Metacognition can be broadly classified into two types based on how it assesses internal states: explicit and implicit metacognition (Nisbett and Wilson, 1977; Hampton, 2001; Persaud et al., 2007; Dienes and Seth, 2010; Yuki et al., 2019; Fleming, 2024). Explicit metacognition is quantified by directly assessing the magnitude of beliefs. It is assumed to require verbal reporting and involves conscious access to one’s internal state. Explicit metacognition depends on working memory and is influenced by cognitive load because it involves verbal reporting (Coutinho et al., 2015; Conte et al., 2023). Implicit metacognition requires an indirect evaluation of beliefs in the form of betting choices. Therefore, it does not require verbal reporting and is often used in animal studies of metacognition (Hampton, 2001; Smith, 2009; Yuki and Okanoya, 2017; Miyamoto et al., 2021).

Furthermore, these two metacognitions are assumed to be functionally distinct. Explicit metacognition is thought to contribute to interindividual decision-making by communicating beliefs through language, whereas implicit metacognition is thought to be important for adaptive behavioral control at the individual level (Frith, 2012; Shea et al., 2014; Heyes et al., 2020). This functional segregation is partially supported by several neuroimaging studies. The medial prefrontal cortex is commonly involved in metacognition with reference to one’s own state (Vaccaro and Fleming, 2018). However, some brain regions such as the lateral prefrontal cortex, which are involved in verbalization processes, are thought to play a role only in explicit metacognition (Fleming and Dolan, 2012). These results suggest that these two metacognitions have distinct functions and that their neural substrates are partially segregated. Considering the expected functional differences that explicit metacognition is involved in interindividual communication and decision-making, while implicit metacognition is involved in adaptive behavioral control at the individual level, it can be predicted that metacognitive efficiency is higher in implicit than in explicit metacognition.

### 1.6 The goal of this study

In the current study, we investigated the differences between explicit and implicit prospective metacognition by providing participants with identical behavioral tasks that differed only in the instructions to alter their metacognitive strategies. We extended the auditory delayed match-to-sample task with bet selection developed by Yuki et al. (2019) with a task that asked for prior beliefs about memory in the form of a bet, and a task that asked for prior beliefs about memory directly. We also varied the level of difficulty for the participants to examine the influence of environmental factors on each metacognition. To quantify the efficiency of each type of metacognition, we estimated M-ratio (Maniscalco and Lau, 2012; Fleming, 2017). M-ratio is an index of metacognitive efficiency based on the signal detection theory, which excludes confidence bias and performance-dependent components. Our results show that metacognitive efficiency increased when participants were asked to rate their confidence in making decisions.

## 2 Materials and methods

### 2.1 Participants

A total of 237 adults participated in this online study through Lancers Inc., a crowdsourcing platform in Japan. Each participant had normal hearing and no history of neurological diseases or absolute pitch. Thirty-three participants (14%) were excluded from the analysis due to either a failure to respond or providing too few responses (exclusion criteria details are in the Analysis section below). The final analysis included 204 participants (83 females, 120 males), aged 19–50 years, with a mean age of 38.5 ± 7.3 years (mean ± SD). All participants provided informed consent before the study. This study was approved by the Doshisha University Ethics Committee for Research Plans Involving Human Subjects (no. 19042 and 23020).

### 2.2 Stimuli

We used tone sequences developed by Yuki et al. (2019) as stimuli to control task difficulty. Each tone sequence had seven distinct tones, each lasting 100 ms and comprising six harmonic components (−6 dB/oct.). The fundamental frequency (F0) of each tone was selected from the following nine frequencies: 440.0, 493.9, 554.4, 622.3, 698.5, 784.0, 880.0, 987.8, and 1108.7 Hz. All sound stimuli were created digitally using MATLAB software (R2014b; MathWorks Inc., Natick, MA, United States) at a 16-kHz sampling rate and 16-bit depth.

### 2.3 Procedure

**Figure 1:**
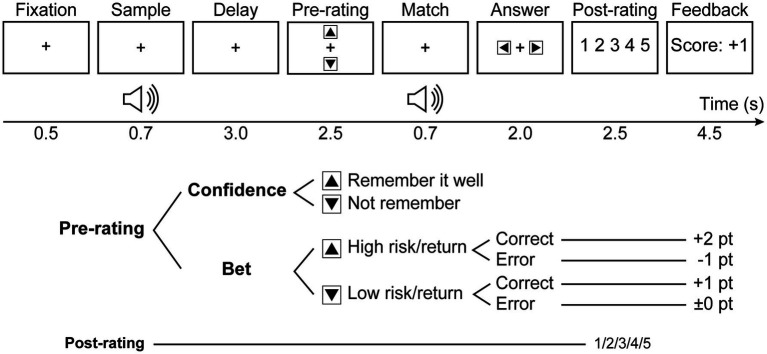
Task sequence. In the bet group, participants were required to select one of two betting options, either high or low risk/return, prior to completing the match during the “pre-rating” phase. In the confidence group, participants were asked to rate their confidence in remembering the first tone during the “pre-rating” phase. After the “answer” phase, both groups rated their confidence in their responses on a 5-point scale at the “post-rating” phase.

To compare explicit and implicit metacognition, we conducted an auditory delayed match-to-sample task with bet selection, extending the study by Yuki et al. (2019). The participants were divided into two groups: confidence and bet (Table 1). As shown in Figure 1, the participants in both groups were presented with two sound stimuli separated by a delay (3.0 s) and asked to judge whether they matched. In the confidence group, the participants were asked to rate their confidence in remembering the first sound. However, in the bet group, participants engaged in bet selection before listening to the second sound to maximize rewards unrelated to money. The bet selection had two options: low-risk/return options, earning one point for a correct answer without any penalty for an incorrect answer, and high-risk/return options, earning two points for a correct answer but losing one point for an incorrect answer. After the match response, the participants evaluated their confidence in their responses on a five-point scale. In short, the pre-rating was evaluated differently across the groups; however, the post-rating was assessed identically for both groups. At the end of each trial, participants received feedback on their responses, with the confidence group receiving information about the correctness of the trial (Correct/Incorrect) and the betting group receiving the points they earned on the trial (Score: ±X point).

**Table 1:** Groups of participants and their details.

Group	Sample size	Instruction	Difficulty
Confidence	*N* = 100	Answer whether the sample stimuli is remembered or not	Easy/Hard (within-participant)
Bet	*N* = 104	Select betting option to maximize score	

The only difference between the groups was the content of the task instructions. Other conditions, such as stimuli and difficulty levels, were the same.

The task was conducted over six sessions, each consisting of 12 trials. The level of difficulty varied between the first three sessions and second three sessions of the experiment. In the easy condition, the Pearson correlation between two sounds was 0.1 ≦ *r* ≦ 0.35, and in the hard condition was 0.7 ≦ *r* ≦ 0.95. The order of difficulty was counterbalanced among the participants. To avoid fixing pre-ratings (Clifford et al., 2008), the participants were instructed not to fixate on a single option in each session.

### 2.4 Analysis

To remove outliers from the behavioral data, we first eliminated trials with no response or a response shorter than 100 ms from any one of the three responses. We excluded participants who provided identical responses across all trials within either difficulty condition or had fewer than 18 valid trials in either difficulty condition, accounting for 14% of all participants.

We analyzed accuracy (the rate of correct responses), d’, score (in the confidence group, we emulated a score in the same way as in the bet group), pre-rating, post-rating for each group, and task difficulty level. d’ is sensitivity based on signal detection theory and represents the ability to discriminate between a signal (a sound identical to the sample stimulus) and noise (a sound different from the sample stimulus) so that d’ can eliminate response bias. For statistical analysis, we used JASP 0.19.1.0 and conducted a two-way mixed analysis of variance (ANOVA) to compare behavioral indices across conditions. We calculated the generalized *η* squared (*η*^2^_G_) as its effect size, and the significance level was set at *α* = 0.05. To evaluate the degree of support for the hypothesis, we ran a Bayesian ANOVA, in which the prior distribution was the default Cauchy distribution. Additionally, we conducted frequentist and Bayesian paired t-tests to investigate the relationship between the pre- and post-ratings. We reported BF_incl_ based on the Bayesian model average and BF_10_. We adopted the alternative hypothesis if the BF was greater than 3, and the null hypothesis if it was less than 1/3.

We calculated the meta-d’/d’ (M-ratio) to estimate metacognitive efficiency from the betting choice and confidence ratings (Maniscalco and Lau, 2012; Fleming, 2017). Meta-d’ is the sensitivity of metacognition with bias removed based on signal detection theory (SDT), and M-ratio is the metacognitive efficiency, removing the effect of task performance on meta-d’. An M-ratio of one indicates that the participant behaves metacognitively and is perfectly optimal.

We estimated M-ratio using a hierarchical Bayesian model. Bayesian analysis offers a robust framework for making inferences from limited data by integrating prior knowledge (Fleming, 2017). Hierarchical Bayesian models utilize the hierarchical structure to “pool” information across participants, allowing for reliable estimation even when the number of trials per individual is limited (Gelman et al., 1995). This pooling mechanism enables stable estimation by leveraging information from other participants, which is a particularly advantageous when within-participant data are sparse. In addition, we extended the model used in previous studies to apply a two-way mixed ANOVA.

For estimating M-ratio, Markov chain Monte Carlo methods (MCMC) used three chains with 10,000 iterations, 1,000 burn-ins and the rest being the default parameters of JAGS (Just Another Gibbs Sampler) v4.3.1. We confirmed that R-hat was <1.001, and tail effective sample size (tail-ESS) was sufficient for the parameters of interest in all models. For the statistical tests, we reported the medians, 95% highest density intervals (HDI), and the probability of direction (pd) to test for the presence or absence of the effect of parameters (Makowski et al., 2019). We accepted the alternative hypothesis if the 95% HDI did not include 0 and the pd. was greater than 95%.

## 3 Results

### 3.1 Behavioral performance

**Figure 2:**
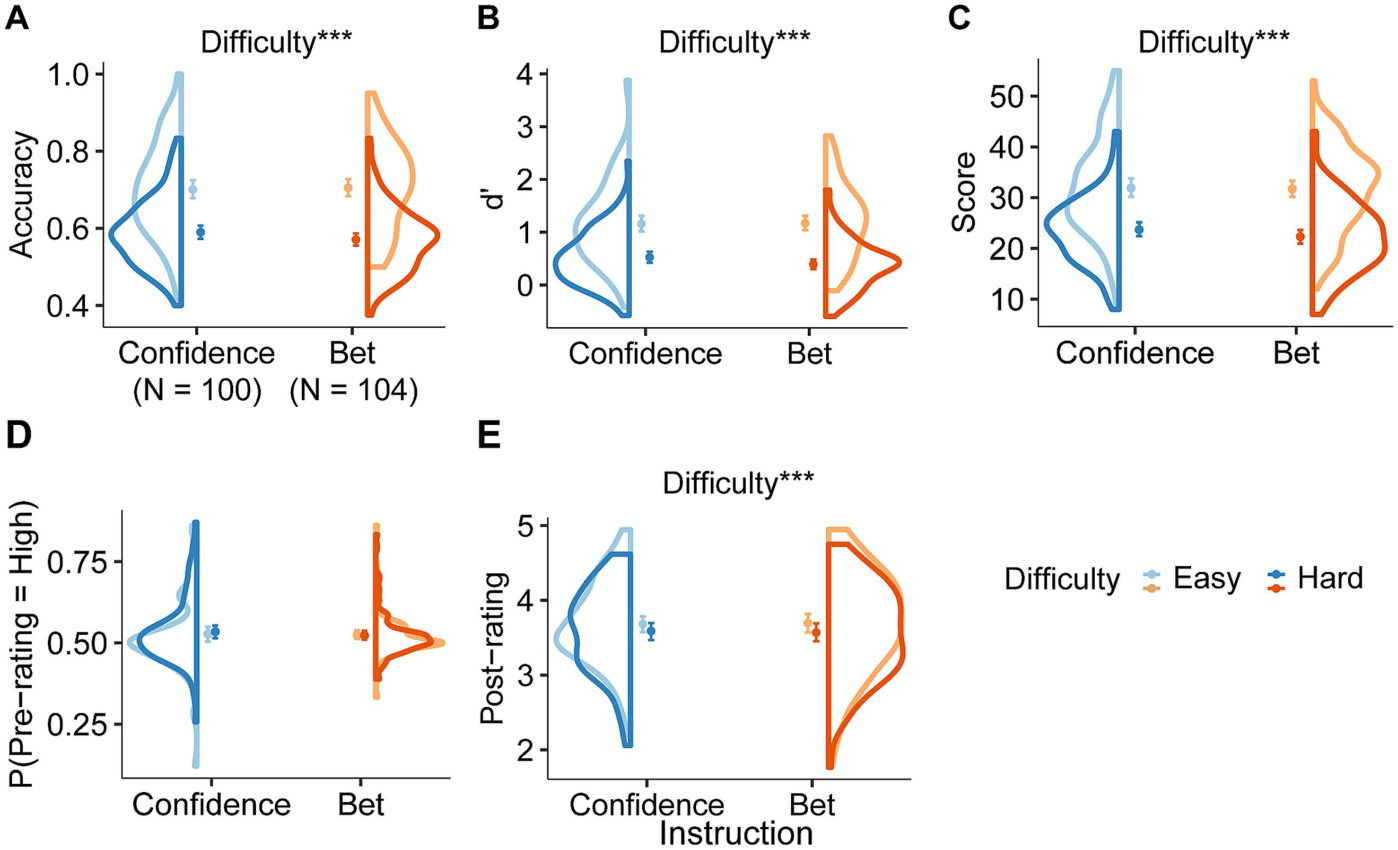
Behavioral performance. Distribution of **(A)** accuracy, **(B)** d’, **(C)** total score, averaged **(D)** pre-rating and **(E)** post-rating of participants for task difficulty in the bet and confidence groups. Accuracy **(A)**, d’ **(B)**, total score **(C)**, and post-rating **(E)** were higher in the easy condition but there was no effect of task instruction. All indices **(A–E)** did not change with the task instruction. Dots indicate the mean. Error bars indicate 95% confidence intervals. Each color (blue or orange) indicates a task instruction, and shades of color indicate difficulty levels. ***p < 0.001, BF > 3.

We first conducted a two-way mixed ANOVA for accuracy, d’, and score in task instruction and difficulty (Table 2). As expected, the average accuracy was higher in the easy condition (Figure 2A, *p* < 0.001, BF_incl_ > 100), but there was no difference between the task instructions and their interaction (*p* > 0.1, BF_incl_ < 1/3). The d’ and total scores also increased in the easy condition (d’ (Figure 2B) and total score (Figure 2C); *p* < 0.001, BF_incl_ > 100), but were not influenced by the task instruction (*p* > 0.1, BF_incl_ < 1/3).

**Table 2:** Results of frequentist/Bayesian two-way mixed ANOVA for accuracy, d’, total score, averaged pre-rating, and post-rating of participants: main effects and interaction between task instruction and difficulty.

Parameters	Cases	*F*	*p*	*η* ^2^_G_	BF_incl_
Accuracy	Instruction	0.347	0.557	0.001	0.172
	**Difficulty**	**221.668**	**<0.001**	**0.261**	**inf**
	Difficulty ✻ Instruction	2.148	0.144	0.003	0.252
d’	Instruction	0.699	0.404	0.002	0.189
	**Difficulty**	**195.034**	**<0.001**	**0.239**	**3.160 × 10** ^**14**^
	Difficulty ✻ Instruction	2.024	0.156	0.003	0.258
Score	Instruction	0.746	0.389	0.002	0.176
	**Difficulty**	**185.177**	**<0.001**	**0.237**	**inf**
	Difficulty ✻ Instruction	0.813	0.368	0.001	0.154
pre-rating	Instruction	0.435	0.510	0.002	0.142
	Difficulty	0.232	0.630	3.202 × 10^−4^	0.083
	Difficulty ✻ Instruction	0.377	0.540	5.205 × 10^−4^	0.013
post-rating	Instruction	0.002	0.968	7.123 × 10^−6^	0.219
	**Difficulty**	**13.840**	**<0.001**	**0.008**	**51.815**
	Difficulty ✻ Instruction	0.353	0.553	2.056 × 10^−4^	0.161

Tests where an alternative hypothesis was accepted are shown in bold.

Next, we examined the effects of instruction and difficulty on the pre- and post-ratings. A two-way mixed ANOVA revealed that the average pre-rating was not affected by task difficulty, instruction, or interaction (Figure 2D, *p* > 0.1, BF_incl_ < 1/3). Average post-confidence was higher in the easy condition (Figure 2E, difficulty: *p* < 0.001; BF_incl_ = 52.161; instruction and interaction: *p* > 0.1, BF_incl_ < 1/3).

### 3.2 Metacognitive efficiency

**Figure 3:**
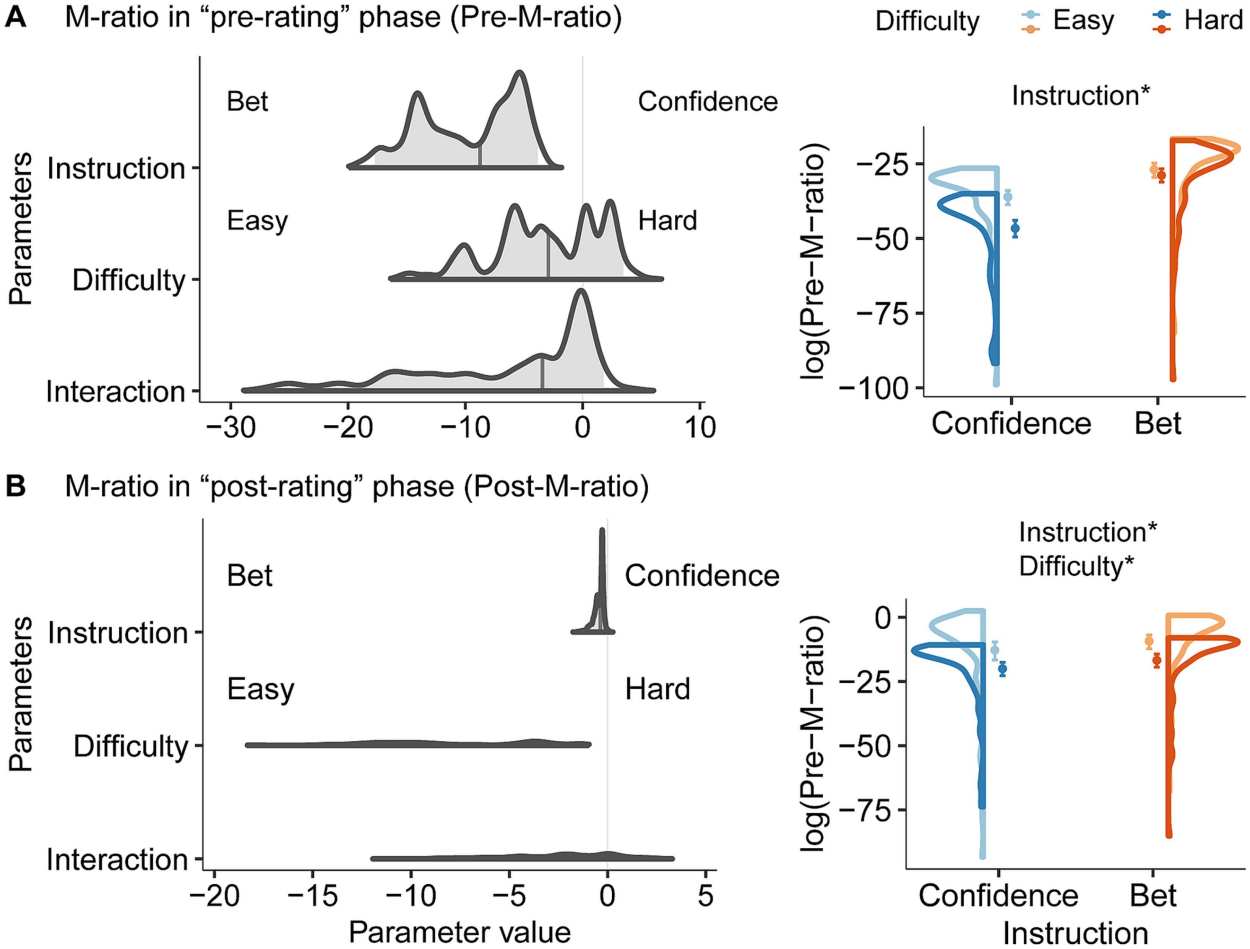
Metacognitive performance in **(A)** “pre-rating” and **(B)** “post-rating” phase. Left: posterior distributions over M-ratio (meta-d’/d’) for task instruction, difficulty, and their interaction. The gray vertical line indicates the median. To help interpret the posterior distribution, log-transformed distribution of the individual data drawn from the group-level parameter of each M-ratio is shown on the right panel. In the “pre-rating” phase, M-ratio was higher for the betting instruction. Similarly, in the “post-rating” phase, M-ratio increased in the betting instruction. Additionally, M-ratio was higher in the easy condition and its effect was greater in the confidence instruction. Dots indicate the mean. Error bars indicate 95% confidence intervals. Each color (blue or orange) indicates a task instruction, and shades of color indicate difficulty levels. *pd. > 95%.

We investigated whether the task instructions influenced metacognitive efficiency. Therefore, we calculated the effect of metacognitive efficiency (M-ratio) on task instruction and difficulty by applying hierarchical Bayesian modeling to the pre- and post-rating data (Table 3). As shown in Figure 3A, the pre-M-ratio increased at the betting instruction (median = −8.755, 95% HDI = [−17.725, −3.827], pd. = 1.000), but there was no main effect of difficulty and their interaction (difficulty: median = −2.922, 95% HDI = [−12.872, 3.460], pd. = 0.643; interaction: median = −3.438, 95% HDI = [−24.914, 1.781], pd. = 0.782). Despite the lack of manipulation in the “post-rating” phase, the post-M-ratio was influenced by task instruction, and difficulty (Figure 3B, instruction: median = −0.366, 95% HDI = [−1.075, −0.110], pd. = 0.992; difficulty: median = −8.924, 95% HDI = [−15.655, −1.314], pd. = 1.000; interaction: median = −1.920, 95% HDI = [−8.351, 1.956], pd. = 0.778). Specifically, the post-M-ratio was higher in the betting instruction and easy conditions. As shown in Figures 3A,B right, the pre- and post-M ratios were <1.

**Table 3:** Summary of Bayesian modeling results for pre- and post-M-ratio: the median and 95% highest density interval (HDI) of posterior distributions, probability of direction (pd), and model convergence metrics like R-hat and tail effective sample size (tail-ESS).

Parameters	Cases	Median	95% HDI	pd	R-hat	Tail-ESS
Pre-M-ratio	**Instruction (Confidence)**	**−8.755**	**[−17.725, −3.827]**	**1.000**	**1.000**	**26843.65**
	Difficulty (Hard)	−2.922	[−12.872, 3.460]	0.643	1.000	25848.45
	Difficulty ✻ Instruction	−3.438	[−24.914, 1.781]	0.782	1.000	26962.36
Post-M-ratio	**Instruction (Confidence)**	**−0.366**	**[−1.075, −0.110]**	**0.992**	**1.000**	**25967.33**
	**Difficulty (Hard)**	**−8.924**	**[−15.655, −1.314]**	**1.000**	**1.000**	**26759.42**
	Difficulty ✻ Instruction	−1.920	[−8.351, 1.956]	0.778	1.000	24951.41

Tests where an alternative hypothesis was accepted are shown in bold.

**Figure 4:**
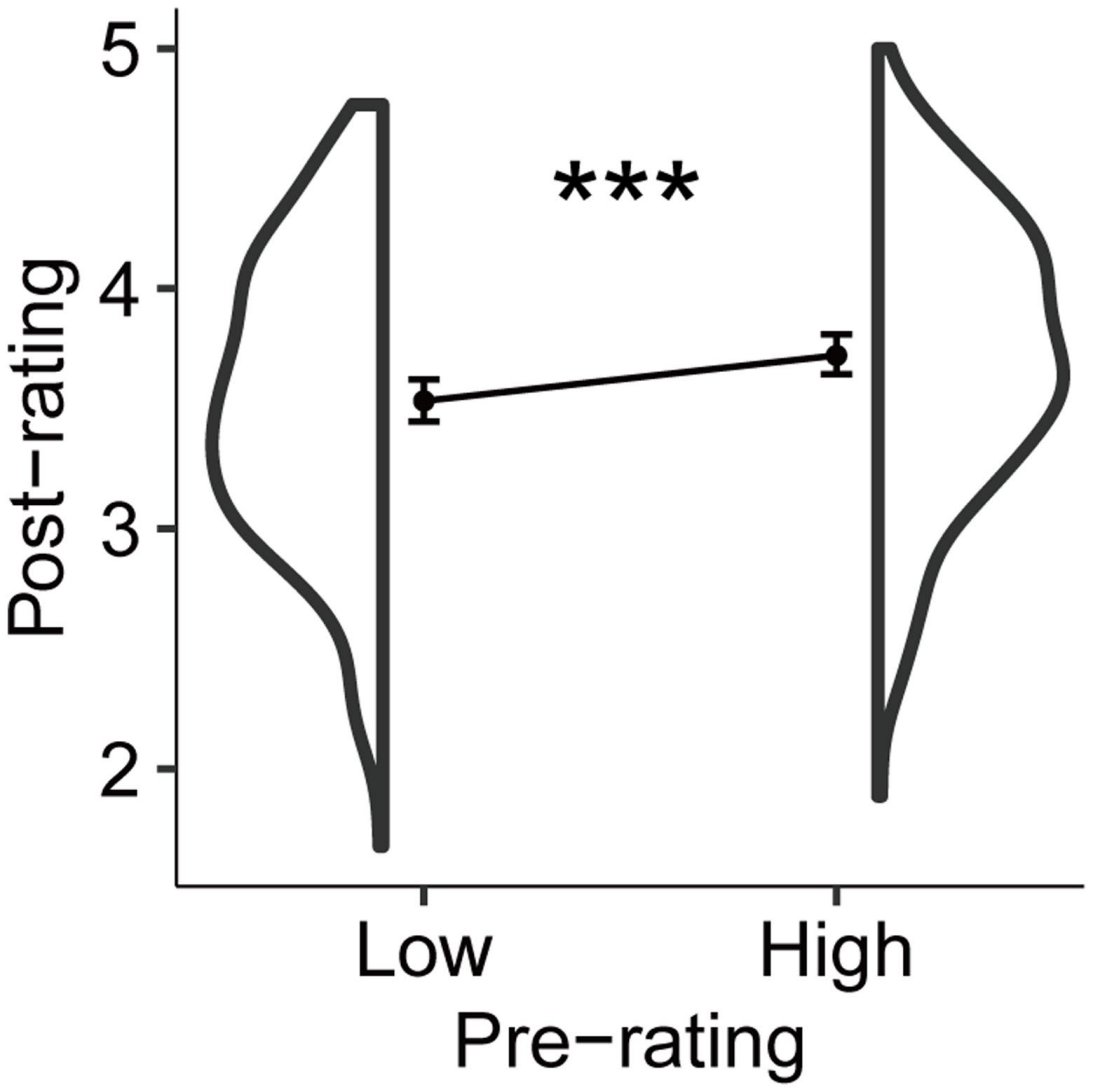
Distribution of averaged post-rating of participants for the pre-rating. The pre-rating was influenced by the post-rating. Dots indicate the mean. Error bars indicate 95% confidence intervals. ***p < 0.001, BF > 3.

We wondered why the instruction manipulated only prospective metacognition, but also showed effects on retrospective metacognition. It was postulated that information derived from the pre-rating stage may have been carried over to the post-rating stage. Consequently, a paired *t*-test was conducted on the post- and pre-rating. The analysis showed that the pre-rating corresponded to the post-rating, that is, if the pre-rating was high, the post-rating was also high [Figure 4, *t*(203) = −8.574, *p* < 0.001, Cohen’s *d* = −0.600, BF_10_ = 2.256 × 10^12^].

## 4 Discussion

The purpose of the present study was to examine whether there is a difference between explicit and implicit metacognition when performing identical tasks with different instructions regarding the strategy of prospective metacognition. Our results showed that both prospective and retrospective metacognition showed increased efficiency when they were provided with betting instructions for prospective metacognition, although the difference in instructions did not affect behavioral performance. Additionally, post-ratings were found to be dependent on pre-ratings. These findings indicate that betting instructions enhance the efficiency of prospective metacognition.

It is noteworthy that metacognitive efficiency appeared to have changed without a corresponding change in behavioral performance. We conducted an exploratory analysis of the pre-rating data and noticed that variations among the participants in the pre-rating appeared to differ for each instruction (Figure 2D). Levene’s test was performed on the variance of pre-ratings for each instruction. The results show that the between-participant variance of the pre-rating in each difficulty condition differed across task instructions [Figure 2D; easy: *F*(1, 202) = 11.021, *p* = 0.002; difficult: *F*(1, 202) = 9.065, *p* = 0.002, Levene’s test with Holm correction]. The low inter-participant variability in the pre-rating of the betting instructions may indicate that behavioral optimization instructions promote prospective metacognition, such as not fixing betting choices regardless of the situation, thereby increasing metacognitive efficiency.

Our results suggest that betting instructions enhance metacognitive efficiency. One possible explanation is that the confidence instruction may induce extra processing, such as the verbal recall of beliefs. In other words, the betting instructions eliminated the need for the process, which may have shortened the cognitive process and thus reduced the room for adding metacognitive errors (Shekhar and Rahnev, 2021; Guggenmos, 2022), resulting in improved metacognitive efficiency. Another explanation is that betting instructions evoked loss aversion tendencies in participants and this improved metacognitive efficiency, as several studies have already suggested (Dienes and Seth, 2010; Fleming and Dolan, 2010; Szczepanowski et al., 2020; Cichoń et al., 2021). However, it was difficult to test these hypotheses directly in this study. Future research should examine the factors that facilitate the efficiency of prospective metacognition through cognitive modeling, including information from past experiences.

Regarding the effect of difficulty, retrospective metacognition was affected by task difficulty, which is consistent with previous studies (Siedlecka et al., 2016, 2019; Luo and Liu, 2023). However, the efficiency of prospective metacognition was not affected by task difficulty. This finding is inconsistent with that of a previous meta-memory study (Koriat, 1997). It is possible that other factors, such as task design, attenuated the effect of difficulty on prospective metacognition. One possible explanation for the lack of a significant discrepancy in metacognitive efficiency between the two difficulty levels is that participants may have subjectively perceived both levels as equally challenging. While the behavioral data showed a substantial difference in performance between the two conditions —with mean accuracy rates of 70% for the easy condition and 60% for the hard condition — it is plausible that these differences did not translate into distinct subjective experiences of difficulty. This hypothesis is supported by evidence indicating that the subjective perception of difficulty has a greater influence on metacognitive efficiency than actual difficulty (Sherman and Seth, 2021). Consequently, the perceived similarity in difficulty levels may have masked the differences in metacognitive efficiency, explaining the absence of significant effects.

This study demonstrates that framing tasks as “betting” can improve metacognitive efficiency. These findings suggest that current training strategies may benefit from incorporating framing techniques into cognitive interventions to develop more efficacious methods. Metacognitive training (MCT), a strategy widely used in clinical settings (Moritz and Woodward, 2007; Moritz et al., 2014), could be further optimized by integrating these framing techniques. The success of framing techniques in other domains, such as education, further underscores the potential of this approach in clinical applications. For instance, Callender et al. (2016) showed that learners with high confidence levels could enhance their metacognitive efficacy through incentivized self-assessment. Nevertheless, additional empirical validation is required to facilitate the practical implementation of the “betting” framework in diverse clinical contexts and to evaluate its generalizability across various settings.

In conclusion, this study sought to determine whether there is a discrepancy in metacognitive efficiency based on confidence ratings and betting. The same task was performed using different metacognition-related instructions, with the only difference being the task instruction content. The results demonstrated that the efficiency of prospective metacognition was enhanced by betting instructions. These findings suggest that metacognitive abilities can be enhanced through the implementation of appropriate metacognitive strategies. In future, it would be beneficial to reveal the differences in cognitive processes between explicit and implicit metacognition to achieve optimal prospective metacognition.

## Data Availability

The datasets and codes for this study are available at https://github.com/hideh1231/MetaCogBet.

## References

[ref1] Arbuckle T. Y. Cuddy L. L. (1969). Discrimination of item strength at time of presentation. J. Exp. Psychol. 81, 126–131. doi: 10.1037/h0027455

[ref2] Callender A. A. Franco-Watkins A. M. Roberts A. S. (2016). Improving metacognition in the classroom through instruction, training, and feedback. Metacogn. Learn. 11, 215–235. doi: 10.1007/s11409-015-9142-6

[ref3] Carpenter J. Sherman M. T. Kievit R. A. Seth A. K. Lau H. Fleming S. M. (2019). Domain-general enhancements of metacognitive ability through adaptive training. J. Exp. Psychol. Gen. 148, 51–64. doi: 10.1037/xge0000505, PMID: 30596440 PMC6390881

[ref4] Cichoń E. Gawęda Ł. Moritz S. Szczepanowski R. (2021). Experience-based knowledge increases confidence in discriminating our memories. Curr. Psychol. 40, 840–852. doi: 10.1007/s12144-018-0011-8

[ref9001] Clifford C. W. G. Arabzadeh E. Harris J. A. (2008). Getting technical about awareness. Trends Cogn. Sci, 12, 54–58. doi: 10.1016/j.tics.2007.11.009 18178511

[ref5] Conte N. Fairfield B. Padulo C. Pelegrina S. (2023). Metacognition in working memory: confidence judgments during an n-back task. Conscious. Cogn. 111:103522. doi: 10.1016/j.concog.2023.103522, PMID: 37087901

[ref6] Coutinho M. V. C. Redford J. S. Church B. A. Zakrzewski A. C. Couchman J. J. Smith J. D. (2015). The interplay between uncertainty monitoring and working memory: can metacognition become automatic? Mem. Cogn. 43, 990–1006. doi: 10.3758/s13421-015-0527-1, PMID: 25971878 PMC4721958

[ref7] Desender K. Van Opstal F. Van den Bussche E. (2017). Subjective experience of difficulty depends on multiple cues. Sci. Rep. 7:44222. doi: 10.1038/srep44222, PMID: 28287137 PMC5347002

[ref8] Dienes Z. Seth A. (2010). Gambling on the unconscious: a comparison of wagering and confidence ratings as measures of awareness in an artificial grammar task. Conscious. Cogn. 19, 674–681. doi: 10.1016/j.concog.2009.09.009 19828331

[ref9] Egner T. Siqi-Liu A. (2024). Insights into control over cognitive flexibility from studies of task-switching. Curr. Opin. Behav. Sci. 55:101342. doi: 10.1016/j.cobeha.2023.101342, PMID: 38186744 PMC10769152

[ref10] Favaretto E. Bedani F. Brancati G. E. De Berardis D. Giovannini S. Scarcella L.. (2024). Synthesising 30 years of clinical experience and scientific insight on affective temperaments in psychiatric disorders: state of the art. J. Affect. Disord. 362, 406–415. doi: 10.1016/j.jad.2024.07.011, PMID: 38972642

[ref11] Fischer H. Huff M. Anders G. Said N. (2023). Metacognition, public health compliance, and vaccination willingness. Proc. Natl. Acad. Sci. USA 120:e2105425120. doi: 10.1073/pnas.2105425120, PMID: 37851676 PMC10614760

[ref12] Flavell J. H. (1979). Metacognition and cognitive monitoring: A new area of cognitive–developmental inquiry. Am. Psychol. 34, 906–911. doi: 10.1037/0003-066x.34.10.906

[ref13] Fleming S. M. (2017). HMeta-d: hierarchical Bayesian estimation of metacognitive efficiency from confidence ratings. Neurosci. Conscious 2017:nix007. doi: 10.1093/nc/nix007, PMID: 29877507 PMC5858026

[ref14] Fleming S. M. (2024). Metacognition and confidence: A review and synthesis. Annu. Rev. Psychol. 75, 241–268. doi: 10.1146/annurev-psych-022423-032425, PMID: 37722748

[ref15] Fleming S. M. Dolan R. J. (2010). Effects of loss aversion on post-decision wagering: implications for measures of awareness. Conscious. Cogn. 19, 352–363. doi: 10.1016/j.concog.2009.11.002, PMID: 20005133 PMC2842936

[ref16] Fleming S. M. Dolan R. J. (2012). The neural basis of metacognitive ability. Philos. Trans. R. Soc. Lond. B Biol. Sci. 367, 1338–1349. doi: 10.1098/rstb.2011.0417, PMID: 22492751 PMC3318765

[ref17] Fleming S. M. Lau H. C. (2014). How to measure metacognition. Front. Hum. Neurosci. 8:443. doi: 10.3389/fnhum.2014.00443, PMID: 25076880 PMC4097944

[ref18] Fleming S. M. Massoni S. Gajdos T. Vergnaud J.-C. (2016). Metacognition about the past and future: quantifying common and distinct influences on prospective and retrospective judgments of self-performance. Neurosci. Conscious 2016:niw018. doi: 10.1093/nc/niw018, PMID: 30356936 PMC6192381

[ref19] Frith C. D. (2012). The role of metacognition in human social interactions. Philos. Trans. R. Soc. Lond. B Biol. Sci. 367, 2213–2223. doi: 10.1098/rstb.2012.0123, PMID: 22734064 PMC3385688

[ref20] Gelman A. Carlin J. B. Stern H. S. Rubin D. B. (1995). Bayesian data analysis: Chapman and Hall/CRC. doi: 10.1201/9780429258411

[ref21] Goupil L. Kouider S. (2016). Behavioral and neural indices of metacognitive sensitivity in preverbal infants. Curr. Biol. 26, 3038–3045. doi: 10.1016/j.cub.2016.09.004, PMID: 27773566 PMC5130696

[ref22] Gourgey A. F. (1998). Metacognition in basic skills instruction. Instr. Sci. 26, 81–96. doi: 10.1023/a:1003092414893

[ref23] Guggenmos M. (2022). Reverse engineering of metacognition. eLife 11:e75420. doi: 10.7554/eLife.75420, PMID: 36107147 PMC9477496

[ref24] Hampton R. R. (2001). Rhesus monkeys know when they remember. Proc. Natl. Acad. Sci. USA 98, 5359–5362. doi: 10.1073/pnas.071600998, PMID: 11274360 PMC33214

[ref25] Hart J. T. (1965). Memory and the feeling-of-knowing experience. J. Educ. Psychol. 56, 208–216. doi: 10.1037/h0022263, PMID: 5825050

[ref26] Hasson-Ohayon I. Avidan-Msika M. Mashiach-Eizenberg M. Kravetz S. Rozencwaig S. Shalev H.. (2015). Metacognitive and social cognition approaches to understanding the impact of schizophrenia on social quality of life. Schizophr. Res. 161, 386–391. doi: 10.1016/j.schres.2014.11.008 25499045

[ref27] Heyes C. Bang D. Shea N. Frith C. D. Fleming S. M. (2020). Knowing ourselves together: the cultural origins of metacognition. Trends Cogn. Sci. 24, 349–362. doi: 10.1016/j.tics.2020.02.007, PMID: 32298621 PMC7903141

[ref28] Katyal S. Fleming S. M. (2024). The future of metacognition research: balancing construct breadth with measurement rigor. Cortex 171, 223–234. doi: 10.1016/j.cortex.2023.11.002, PMID: 38041921 PMC11139654

[ref29] Koriat A. (1997). Monitoring one’s own knowledge during study: A cue-utilization approach to judgments of learning. J. Exp. Psychol. Gen. 126, 349–370. doi: 10.1037/0096-3445.126.4.349

[ref30] Luo T. Liu C. (2023). The impact of feedback on metacognition: enhancing in easy tasks, impeding in difficult ones. Conscious. Cogn. 116:103601. doi: 10.1016/j.concog.2023.103601, PMID: 37951007

[ref31] Makowski D. Ben-Shachar M. S. Chen S. H. A. Lüdecke D. (2019). Indices of effect existence and significance in the Bayesian framework. Front. Psychol. 10:2767. doi: 10.3389/fpsyg.2019.02767, PMID: 31920819 PMC6914840

[ref32] Maniscalco B. Lau H. (2012). A signal detection theoretic approach for estimating metacognitive sensitivity from confidence ratings. Conscious. Cogn. 21, 422–430. doi: 10.1016/j.concog.2011.09.021, PMID: 22071269

[ref33] Martiadis V. Pessina E. Raffone F. Iniziato V. Martini A. Scognamiglio P. (2023). Metacognition in schizophrenia: A practical overview of psychometric metacognition assessment tools for researchers and clinicians. Front. Psych. 14:1155321. doi: 10.3389/fpsyt.2023.1155321, PMID: 37124248 PMC10133516

[ref34] Miyamoto K. Trudel N. Kamermans K. Lim M. C. Lazari A. Verhagen L.. (2021). Identification and disruption of a neural mechanism for accumulating prospective metacognitive information prior to decision-making. Neuron 109, 1396–1408.e7. doi: 10.1016/j.neuron.2021.02.024, PMID: 33730554 PMC8063717

[ref35] Moritz S. Andreou C. Schneider B. C. Wittekind C. E. Menon M. Balzan R. P.. (2014). Sowing the seeds of doubt: a narrative review on metacognitive training in schizophrenia. Clin. Psychol. Rev. 34, 358–366. doi: 10.1016/j.cpr.2014.04.004, PMID: 24866025

[ref36] Moritz S. Woodward T. S. (2007). Metacognitive training in schizophrenia: from basic research to knowledge translation and intervention. Curr. Opin. Psychiatry 20, 619–625. doi: 10.1097/YCO.0b013e3282f0b8ed, PMID: 17921766

[ref37] Nelson T. O. Louis N. (1990). “Metamemory: a theoretical framework and new findings” in Psychology of learning and motivation. ed. Bower G. H. (Elsevier), 125–173. doi: 10.1016/s0079-7421(08)60053-5

[ref38] Nisbett R. E. Wilson T. D. (1977). Telling more than we can know: verbal reports on mental processes. Psychol. Rev. 84, 231–259. doi: 10.1037/0033-295X.84.3.231

[ref39] Pereira M. Faivre N. Iturrate I. Wirthlin M. Serafini L. Martin S.. (2020). Disentangling the origins of confidence in speeded perceptual judgments through multimodal imaging. Proc. Natl. Acad. Sci. USA 117, 8382–8390. doi: 10.1073/pnas.1918335117, PMID: 32238562 PMC7165419

[ref40] Persaud N. McLeod P. Cowey A. (2007). Post-decision wagering objectively measures awareness. Nat. Neurosci. 10, 257–261. doi: 10.1038/nn1840, PMID: 17237774

[ref41] Qiu L. Su J. Ni Y. Bai Y. Zhang X. Li X.. (2018). The neural system of metacognition accompanying decision-making in the prefrontal cortex. PLoS Biol. 16:e2004037. doi: 10.1371/journal.pbio.2004037, PMID: 29684004 PMC5933819

[ref42] Rouy M. de Gardelle V. Reyes G. Sackur J. Vergnaud J. C. Filevich E.. (2022). Metacognitive improvement: disentangling adaptive training from experimental confounds. J. Exp. Psychol. Gen. 151, 2083–2091. doi: 10.1037/xge0001185 35157481

[ref43] Shea N. Boldt A. Bang D. Yeung N. Heyes C. Frith C. D. (2014). Supra-personal cognitive control and metacognition. Trends Cogn. Sci. 18, 186–193. doi: 10.1016/j.tics.2014.01.006, PMID: 24582436 PMC3989995

[ref44] Shekhar M. Rahnev D. (2021). The nature of metacognitive inefficiency in perceptual decision making. Psychol. Rev. 128, 45–70. doi: 10.1037/rev0000249, PMID: 32673034 PMC7883626

[ref45] Sherman M. T. Seth A. (2021). Effects of expected task difficulty on metacognitive confidence and multitasking. [Epubh ahead of preprint]. doi: 10.31234/osf.io/3gfp2

[ref46] Siedlecka M. Paulewicz B. Wierzchoń M. (2016). But I was so sure! Metacognitive judgments are less accurate given prospectively than retrospectively. Front. Psychol. 7:218. doi: 10.3389/fpsyg.2016.00218, PMID: 26925023 PMC4759291

[ref47] Siedlecka M. Skóra Z. Paulewicz B. Fijałkowska S. Timmermans B. Wierzchoń M. (2019). Responses improve the accuracy of confidence judgements in memory tasks. J. Exp. Psychol. Learn. Mem. Cogn. 45, 712–723. doi: 10.1037/xlm0000608, PMID: 29999396

[ref48] Smith J. D. (2009). The study of animal metacognition. Trends Cogn. Sci. 13, 389–396. doi: 10.1016/j.tics.2009.06.009 19726218

[ref49] Szczepanowski R. Cichoń E. Pasieczna A. H. Gawęda Ł. Rosińczuk J. (2020). Monetary incentives increase metacognitive confidence in source memory performance in patients with schizophrenia. Front. Psych. 11:725. doi: 10.3389/fpsyt.2020.00725, PMID: 32848910 PMC7403207

[ref50] Vaccaro A. G. Fleming S. M. (2018). Thinking about thinking: a coordinate-based meta-analysis of neuroimaging studies of metacognitive judgements. Brain Neurosci. Adv. 2:2398212818810591. doi: 10.1177/2398212818810591, PMID: 30542659 PMC6238228

[ref51] van den Berg R. Anandalingam K. Zylberberg A. Kiani R. Shadlen M. N. Wolpert D. M. (2016). A common mechanism underlies changes of mind about decisions and confidence. eLife 5:e12192. doi: 10.7554/eLife.12192, PMID: 26829590 PMC4798971

[ref52] Wells A. (2011). Metacognitive therapy for anxiety and depression. New York, NY: Guilford Publications.

[ref53] Yuki S. Nakatani H. Nakai T. Okanoya K. Tachibana R. O. (2019). Regulation of action selection based on metacognition in humans via a ventral and dorsal medial prefrontal cortical network. Cortex 119, 336–349. doi: 10.1016/j.cortex.2019.05.001, PMID: 31181421

[ref54] Yuki S. Okanoya K. (2017). Rats show adaptive choice in a metacognitive task with high uncertainty. J. Exp. Psychol. Anim. Learn. Cogn. 43, 109–118. doi: 10.1037/xan0000130 28045298

[ref55] Zohar A. Barzilai S. (2013). A review of research on metacognition in science education: current and future directions. Stud. Sci. Educ. 49, 121–169. doi: 10.1080/03057267.2013.847261

